# Multi-robot cooperative autonomous exploration via task allocation in terrestrial environments

**DOI:** 10.3389/fnbot.2023.1179033

**Published:** 2023-06-05

**Authors:** Xiangda Yan, Zhe Zeng, Keyan He, Huajie Hong

**Affiliations:** ^1^Laboratory of Unmanned Combat Systems, National University of Defense Technology, Changsha, China; ^2^Rescue & Salvage Department, Navy Submarine Academy, Qingdao, China

**Keywords:** cooperative autonomous exploration, frontier, Mean-Shift, path planning, task allocation, multi-robot

## Abstract

Cooperative autonomous exploration is a challenging task for multi-robot systems, which can cover larger areas in a shorter time or path length. Using multiple mobile robots for cooperative exploration of unknown environments can be more efficient than a single robot, but there are also many difficulties in multi-robot cooperative autonomous exploration. The key to successful multi-robot cooperative autonomous exploration is effective coordination between the robots. This paper designs a multi-robot cooperative autonomous exploration strategy for exploration tasks. Additionally, considering the fact that mobile robots are inevitably subject to failure in harsh conditions, we propose a self-healing cooperative autonomous exploration method that can recover from robot failures.

## 1. Introduction

Robotic technology has advanced rapidly in recent years, and there have been many developments in various fields of robot technology. However, one of the challenges that researchers still face is the map-building process for autonomous mobile robots. One of the main challenges in map-building for autonomous mobile robots is the complexity of the environment. Complicated environments, such as indoor spaces with many obstacles or outdoor environments with uneven terrain, can make it difficult for the robot to explore autonomously (Liu and Nejat, 2013; Qiu and Kermani, 2021).

However, we hope that robots have the potential to replace humans in search and rescue operations in deplorable conditions, such as after natural disasters or in hazardous environments. One of the key capabilities required for robots in these situations is the ability to build maps of unknown environments autonomously. Multi-robot cooperative autonomous exploration can be more efficient than single-robot exploration but also poses many difficulties (Liu and Nejat, 2016).

Efficient cooperative methods are essential for multi-robot systems to explore unknown environments effectively. Without an efficient cooperative method, robots may interfere with each other, resulting in a decrease in efficiency and potentially even system failure (Casper and Murphy, 2003). One of the main challenges in designing efficient cooperative methods for multi-robot systems is task allocation. Each robot must be assigned tasks that are suitable for its capabilities and that contribute to the overall goal of the system.

Maps come in a variety of types, including topological maps, feature maps, occupancy grid maps, and more. Laumond proposed the topological map. Nonetheless, there is no use of the topological model to cope with measure inaccuracy (Laumond, 1983; Chatila and Laumond, 1985; Kuipers and Byun, 1991; Konolige, 1997). A statistical approach derived from Bayes's theorem is quick and efficient, which analyzes the issue of creating occupancy grid maps from diverse sources of information (Moravec, 1988).

Thrun and Bücken offered a method for autonomously controlling a mobile robot outfitted with sonar sensors that blend two paradigms: grid-based and topological. While this method offers a useful approach for mobile robot control, there are limitations to its applicability. One of the main limitations of Thrun and Bücken's method is the assumption that all walls in the environment are parallel or perpendicular to one another. This assumption is only appropriate in environments with regular shapes, such as rectangular rooms or corridors. In environments with irregular shapes or non-orthogonal walls, this assumption may lead to errors, which can affect its ability to explore unknown environments effectively (Thrun and Bücken, 1996).

Frontier was first put forth by Yamauchi (1997). The extraction of frontiers at the intersection of free grids and unknown grids in occupancy grid maps is a useful approach because it enables the robot to identify areas in the environment that have not yet been explored. The robot is then navigated to the closest frontier in order to complete its goal of autonomous exploration. Subsequently, Brian Yamauchi expanded this approach to multi-robot cooperative autonomous exploration (Yamauchi, 1998). While this approach has several advantages, such as enabling robots to explore new areas of the environment efficiently and effectively, there are also limitation. One of the main limitations is that there is no coordination between robots and no centralized control over the exploration process. This can lead to low productivity, as robots may spend significant amounts of time exploring areas that have already been explored while neglecting other areas that have not yet been explored.

As one of the most challenging environments, underwater environment is a kind of open area (Xanthidis et al., 2022). Before carrying out relevant research, we also focused on the relative solutions in this field. Normally, in the task of explore underwater environments, the AUVs perform a pre-planned coverage path for exploration. This kind of exploration mode for unknown environments is not suitable for scenarios that require to avoid obstacles and decide on optimal or nearly optimal exploration routes in real time, such as indoor surroundings (Ling et al., 2023).

Considering the cost of robot movement and the utility of target point, Simmons made several attempts to reduce the overall time of cooperative autonomous exploration. Specifically, Simmons proposed a method that allocates target points to robots based on their location and the utility of the target point to other robots. The utility of the target point to other robots decreases once it has been allocated to a particular robot, which ensures that robots are allocated target points that are most useful to them. Although this method drastically cuts down on time, there is still a large area for optimization (Burgard et al., 2000; Simmons et al., 2000).

Yan sampled on the Voronoi diagram to build a different topological graph for every robot, but this method will lead to a short-duration disturbance among each other (Yan et al., 2010). Topiwala proposed a method called Wave-front Frontier Detector (WFD) to detect frontiers (Topiwala et al., 2018). It is on the basis of the breadth-first search (BFS) methods, only detects known areas. This key difference reduces the time complexity. Finally, a single robot is used to explore an environment in ROS.

Yu proposed an exploration method based on a multi-robot multi-target potential field for band-limited communications systems (Yu et al., 2021). Robots are assigned to targets by introducing the potential field function, to avoid overlap of trajectory and improve performance. Meanwhile, various intelligent methods have been more and more widely applied and their favorable prospects are emerging (Chen et al., 2019, 2020).

This paper proposes a step-by-step approach to multi-robot cooperative autonomous exploration based on frontiers. The frontier-based exploration involves identifying the frontiers (the boundary between the known and unknown areas of the environment) and assigning robots to explore these frontiers (Senarathne et al., 2013). This approach is effective because it ensures that the robots explore new areas of the environment and avoid revisiting areas that have already been explored. Another important aspect of multi-robot cooperative autonomous exploration is the ability to handle robot failure. This can be achieved through the use of proposed self-healing algorithms in this paper, where the remaining robots re-allocate the failed robot's tasks and continue the exploration task. The self-healing cooperative autonomous exploration method improves the robustness of the algorithm and maintenance efficiency significantly.

## 2. Preliminary

The occupancy grid map is a widely used technique in autonomous mobile robot navigation due to its simplicity and ease of implementation. It involves dividing the environment into a grid of cells and assigning values to each cell to represent the occupancy of that cell. Each cell has only two states(*s*), *s* = 1 indicates that the cell is free, and *s* = 0 indicates that the cell is occupied. While the occupancy grid map is a useful technique, sensor information can contain errors and uncertainties that can affect the accuracy of the map. As a result, it is more appropriate to use probability to represent the state of the map, rather than binary occupancy values. This is known as the probabilistic occupancy grid map. For a grid *m*_*i,j*_ in the map, use *p*(*m*_*i,j*_) to represent the probability that the grid is occupied, which can be called belief (*bel*_*t*_(*m*_*i,j*_)), use $p\left({\overset{\bar{\;}}{m}}_{i,j}\right)$ or 1 − *p*(*m*_*i,j*_) to represent the probability that the grid is free. For the grid that has never been observed by the robot, the initial probability *p*(*m*_*i,j*_) is 0.5.

The whole map is divided into grids. Therefore, the problem is transformed into solving each grid independently. The probability that a grid is occupied is recorded as *bel*(*m*_*i,j*_) = *p*(*m*_*i,j*_|*x*_*t*_, *z*_*t*_). In order to lessen the effect of observation error, the status space of the grid map can be determined by the threshold setting method (Chen et al., 2020).


$$\begin{array}{l} \begin{array}{ccc} p\left(m_{i,j}\right)=1\; & \;bel\left(m_{i,j}\right)>0.6 & Occupied\;Grid\\ p\left(m_{i,j}\right)=0.5 & 0.4\leq bel\left(m_{i,j}\right)\leq 0.6 & Unkown\;Grid\\ p\left(m_{i,j}\right)=0\; & bel\left(m_{i,j}\right)<0.4 & Free\;Grid \end{array} \end{array}$$

Belief function of each grid in occupancy grid maps can be obtained from Bayes's theorem (Moravec, 1988):


$$\begin{array}{l} \begin{array}{c} bel_{t}\left(m_{i,j}\right)=1-\frac{1}{1+exp\left(l_{t}\right)}\\ l_{t}=l_{t-1}+l_{inv}-l_{0}\\ Notes:l_{t-1}=log\left[\frac{bel_{t-1}\left(m_{i,j}\right)}{bel_{t-1}\left({\bar{m}}_{i,j}\right)}\right],l_{inv}=log\left[\frac{p\left(m_{i,j}|x_{t},z_{t}\right)}{p\left({\bar{m}}_{i,j}|x_{t},z_{t}\right)}\right],\\ l_{0}=log\left[\frac{p\left(m_{i,j}\right)}{p\left({\bar{m}}_{i,j}\right)}\;\right] \end{array} \end{array}$$

The updating formula of an occupancy grid map by multiple robots is as follows on the basis of the updating formula by a single robot:


$$\begin{array}{l} l_{t}=l_{t-1}\;+\;l_{inv}^{1}\;+\;l_{inv}^{2}\;+\cdots +\;l_{inv}^{i}\;+\cdots +\;l_{inv}^{n}\;-\;l_{0}\\ Notes:\;l_{inv}^{i}=log\left[\frac{p\left(m_{i,j}|x_{t}^{i},z_{t}^{i}\right)}{p\left({\bar{m}}_{i,j}|x_{t}^{i},z_{t}^{i}\right)}\;\right] \end{array}$$

**Figure 1:**
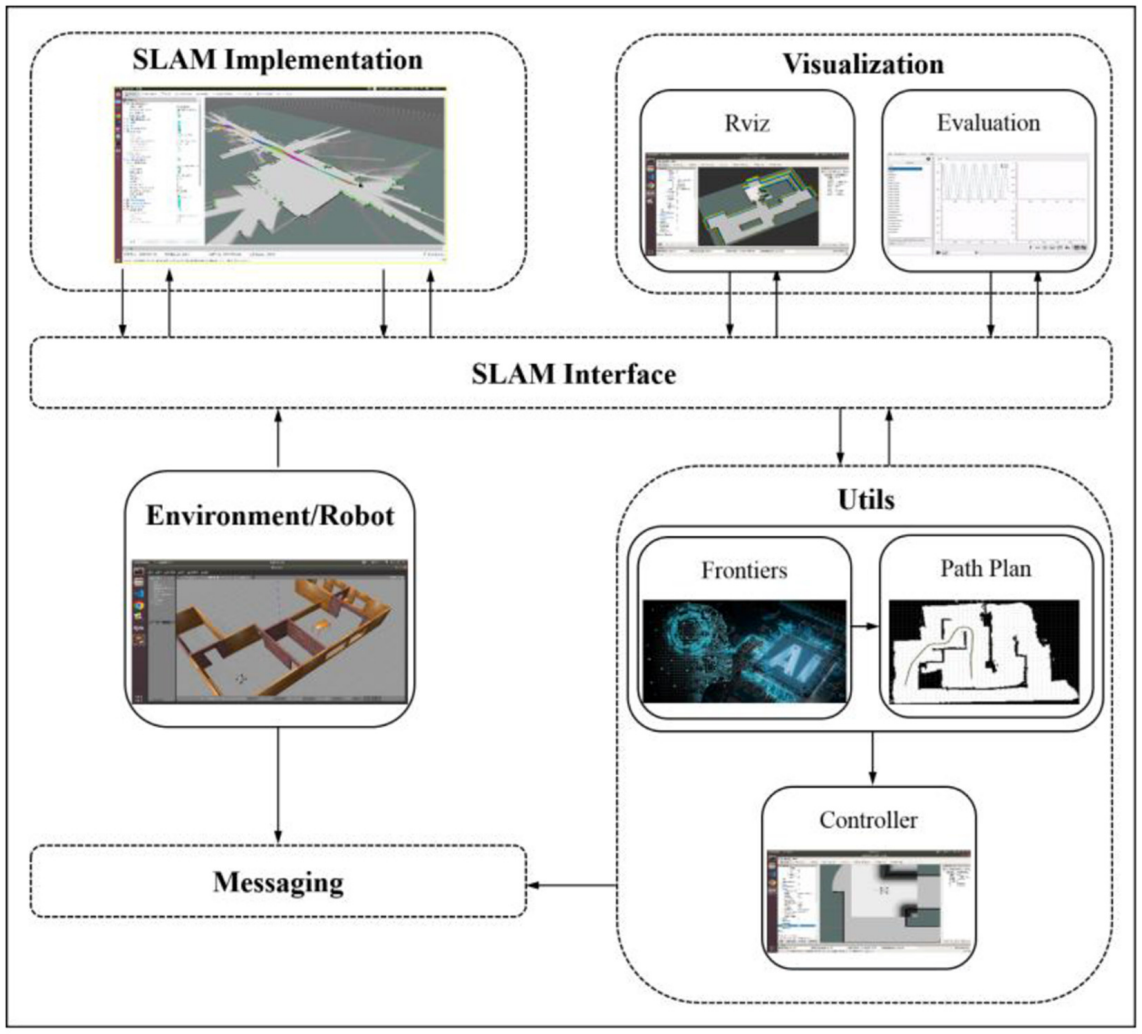
Autonomous exploration.

A general frame of the cooperative autonomous exploration algorithm is constructed here, as shown in Figure 1. The SLAM module is responsible for updating the map based on sensor data, the Frontier module is responsible for calculating target navigation points for the mobile robot based on the map, and the Path Planning module is responsible for planning the path for the mobile robot. Next, we will expatiate the specific design of each part of the whole system.

The structure of this paper is as follows. In the second section, some preliminary research on that is done, and the principle of map fusion in multi-robot collaborative mapping and map merging has been introduced in detail. In Section 3.1, candidate target points are generated to guide the robot's exploration and ensure that it covers as much of the environment as possible. The approach to generating candidate target points is based on frontier grouping and clustering, which can improve the distribution of robots, avoid trajectory overlap and reduce redundancy. Section 3.2 describes the task as an integer programming problem and gives a solution. A self-healing cooperative autonomous exploration method is further proposed in case of a robot failure, which improves robustness and maintenance efficiency significantly. In Section 3.3, a path optimization algorithm is proposed to greatly shorten path length compared with initial paths. In the fourth section, the simulation experiments are carried out and the experimental results are analyzed. Experiments results proved the method is feasible.

## 3. The proposed algorithm

### 3.1. Frontiers grouping and clustering

**Figure 2:**
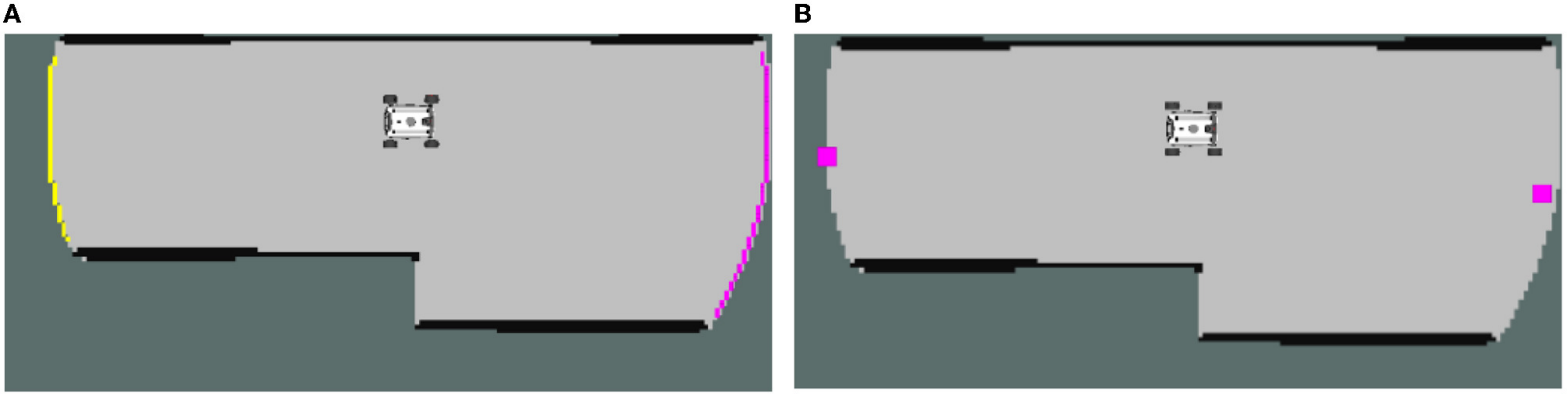
**(A)** Frontier grouping; **(B)** Frontier clustering.

Frontier detection is an important step in autonomous mobile robot exploration, as it identifies areas of the environment that have not yet been explored (Senarathne et al., 2013). However, assigning frontiers to robots without any further processing may not be efficient for collaborative exploration, as it can lead to interference and redundancy. Therefore, frontier grouping is proposed to divide frontiers into groups in order to improve the distribution of robots and reduce redundancy. The algorithm is shown in Table 1. Figure 2A shows the comparison before and after grouping, different colors represent different groups. This algorithm can effectively distinguish different exploration areas.

**Table 1:** Algorithm—Frontiers grouping.

**Algorithm 1: Frontiers grouping**
Input: Frontiers *F* = {*f*_1_, *f*_2_, ⋯ , *f*_*n*_}, Threshold ε
Output: Groups *G* = {*g*_1_, *g*_2_, ⋯ , *g*_*m*_}
1: while *F* is not empty:
2: Initialize two queues: Open = [], Close = []
3: Open.push(*f*_1_), Frontiers.pop(*f*_1_)
4: while Open is not empty:
5: temp_point = Open.pop()
6: for each *f* in *F* do :
7: if |*f* − *temp* < *uscore* > *point*| < ε do :
8: Open.push(*f*)
9: end for
10: Close.push(temp_point)
11 end while
12: *G*.push (Close)
13: end while

Then, Mean-Shift was performed on the frontiers in different groups separately, which improves the efficiency of autonomous exploration. Mean-Shift is a hill-climbing algorithm that involves shifting the kernel iteratively to a higher density region until convergence. Each shift is defined by a mean shift vector, which is the weighted average of the distances between the point and its neighbors in the kernel. The mean shift vector is used to update the location of the kernel until it converges to the mode. For *m* sample points in two-dimensional space *R*^2^, select any point *x* in the space, and the general form of the Mean-Shift vector is defined as:


$$\begin{array}{l} M_{h}=\frac{1}{K}\underset{x_{i}\in S_{k}}{\sum }\left(x_{i}-x\right) \end{array}$$

*S*_*k*_ is defined as a circular area with a radius of *h* and a center of *x* in two-dimensional space, that is, a collection of points *y* meeting the following relationships. *k* indicates that *k* points fall into the region *S*_*k*_ among all sampling points.


$$\begin{array}{l} S_{k}\left(x\right)=\left\{y:{\left(y-x\right)}^{T}\left(y-x\right)<h^{2}\right\} \end{array}$$

The mean shift vector always points toward the direction of the maximum increase in density. At every iteration, the kernel is shifted to the centroid or the mean of the points within it. At convergence, there will be no direction in which a shift can accommodate more points inside the kernel. And Mean-Shift can automatically determine the number of categories. The algorithm is shown in Table 2. Figure 2B shows results over frontier clustering using Mean-Shift. This algorithm can extract more representative feature points from the original boundary points, which greatly reduces the computational load of task allocation and improves the efficiency of task allocation.

**Table 2:** Algorithm—Mean-Shift.

**Algorithm 2: Mean-Shift**
Input: Dataset *D* = {*p*_1_, *p*_2_, ⋯ , *p*_*n*_}, Radius *r*, Threshold δ, ξ
Output: Cluster centroids *C* = {*q*_1_, *q*_2_, ⋯ , *q*_*m*_}
1: for *i* = 1 ⋯ *n* do :
2: while True do :
3: $S_{k}\left(x\right)=\left\{x:{\left(p_{i}-x\right)}^{T}\left(p_{i}-x\right)<h^{2}\right\}$
4: *C* = *C*_*x*_*j*_ ∈ *S*_*k*_(*x*)_ ∪ *p*_*i*_
5: *f*_*p*_*i*__ = ∑|*S*_*k*_(*x*) |
6: $C_{h}=\frac{1}{K}\underset{x_{j}\in S_{k}}{\sum }\left(x_{j}-p_{i}\right)$
7: *x* = *x* + *C*_*h*_
8: if $|C_{h}^{new}-C_{h}^{old}|<\delta$ do :
9: break
10: end while
11: for k = 1 ⋯ |*C*| do :
12: if |*x*_*k*+1_ − *x*_*k*_| < ξ do :
13: *q*_*k*_ = *q*_*k*_ ∪ *q*_*k* + 1_
14: *f*_*k*_ = *f*_*k*_+*f*_*k* + 1_
15: else
16: k++
17: end for
18: end for
19: for *i* = 1 ⋯ *n* do :
20: *C* = *C*_*k*_(*p*_*i*_ ∈ *Max*(*f*_*k*_))
21: end for

### 3.2. Task allocation

Task allocation determines which robot is responsible for exploring which area of the environment. But before allocating tasks, it is necessary to calculate the cost matrix from the robot to the cluster, which represents the distance or cost for each robot to reach each cluster. One approach to calculating the cost matrix is to use the path planning algorithm to obtain the path length.

In this paper, the task allocation problem is described as the following mathematical problem. Firstly, we need to expand the cost matrix *C*^*n* × *m*^ into a square matrix *C*^max(*n, m*) × max(*n, m*)^. (If *n* > *m*, other elements of the square matrix are set to infinity, if *n* < *m*, other elements of the square matrix are set to zero).


$$\begin{array}{l} C^{max\left(n,m\right)\times max\left(n,m\right)}=\{\begin{array}{c} \begin{array}{cc} \left[\begin{array}{cc} C^{n\times m} & Inf^{n\times \left(n-m\right)} \end{array}\right] & n>m \end{array}\\ \begin{array}{cc} C^{n\times m} & \;n=m \end{array}\\ \begin{array}{cc} \left[\begin{array}{c} C^{n\times m}\\ 0^{\left(m-n\right)\times m} \end{array}\right] & n<m \end{array} \end{array}\\ \begin{array}{c} \;minJ=\min\sum_{i=1}^{\max\left(n,m\right)}\sum_{j=1}^{\max\left(n,m\right)}c_{ij}a_{ij} \end{array}\\ \;\begin{array}{cc} s.t, & \sum_{i=1}^{\max\left(n,m\right)}a_{ij}=1,i=1,2,\cdots ,max\left(n,m\right);\\ 1 & \sum_{j=1}^{\max\left(n,m\right)}a_{ij}=1,j=1,2,\cdots ,max\left(n,m\right)\\ 1 & a_{ij}=0\;or\;1,i,j=1,2,\cdots ,max\left(n,m\right) \end{array} \end{array}$$

**Figure 3:**
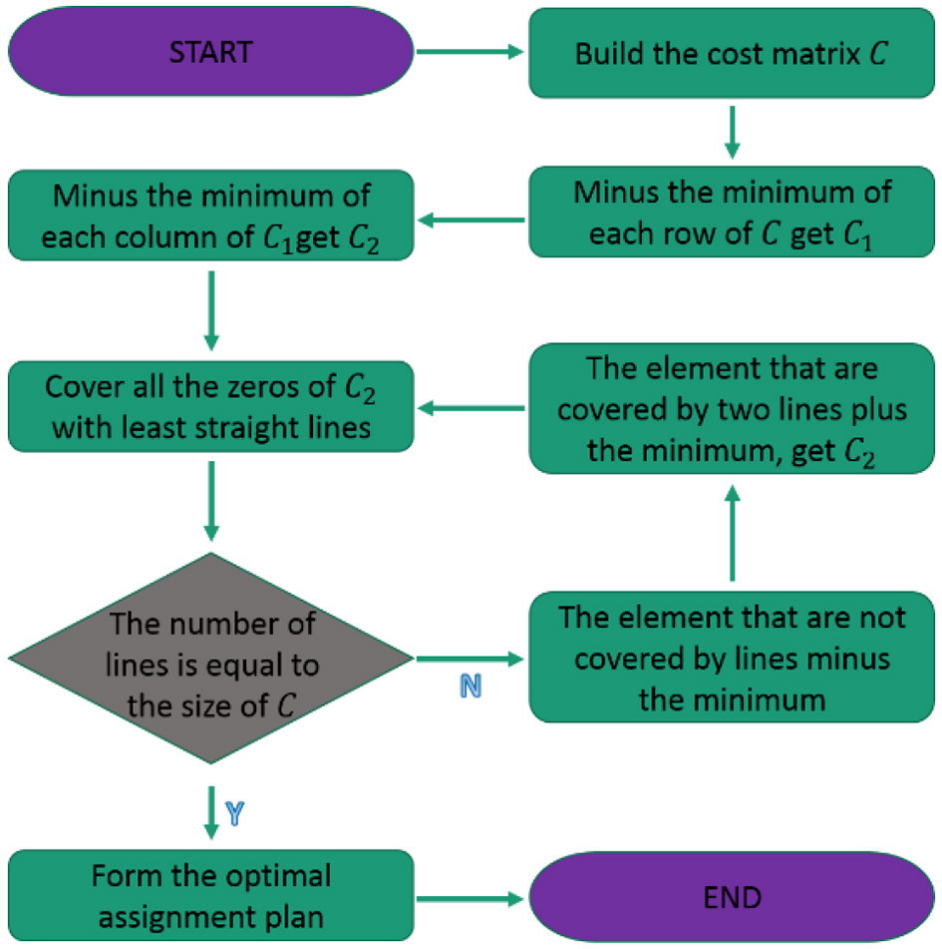
Hungarian algorithm flowchart.

To maximize the efficiency of multi-robot cooperative autonomous exploration, we use the Hungarian algorithm to realize the task allocation problem. The flowchart of solving the problem above by the Hungarian algorithm (Jonker and Volgenant, 1986) is divided in seven steps, as shown in Figure 3.

1. Create a matrix of costs for each task.
2. Subtract the smallest value in each row from all the elements in that row.
3. Subtract the smallest value in each column from all the elements in that column.
4. Draw lines through the rows and columns so that all zeros are covered and the minimum number of lines is used.
5. If the number of lines is equal to the number of tasks, an optimal solution has been found. If not, proceed to step 6.
6. Determine the smallest uncovered value in the matrix. Subtract this value from all uncovered values and add it to all elements that are covered by two lines.
7. Repeat steps 4–6 until an optimal solution is found.

**Figure 4:**
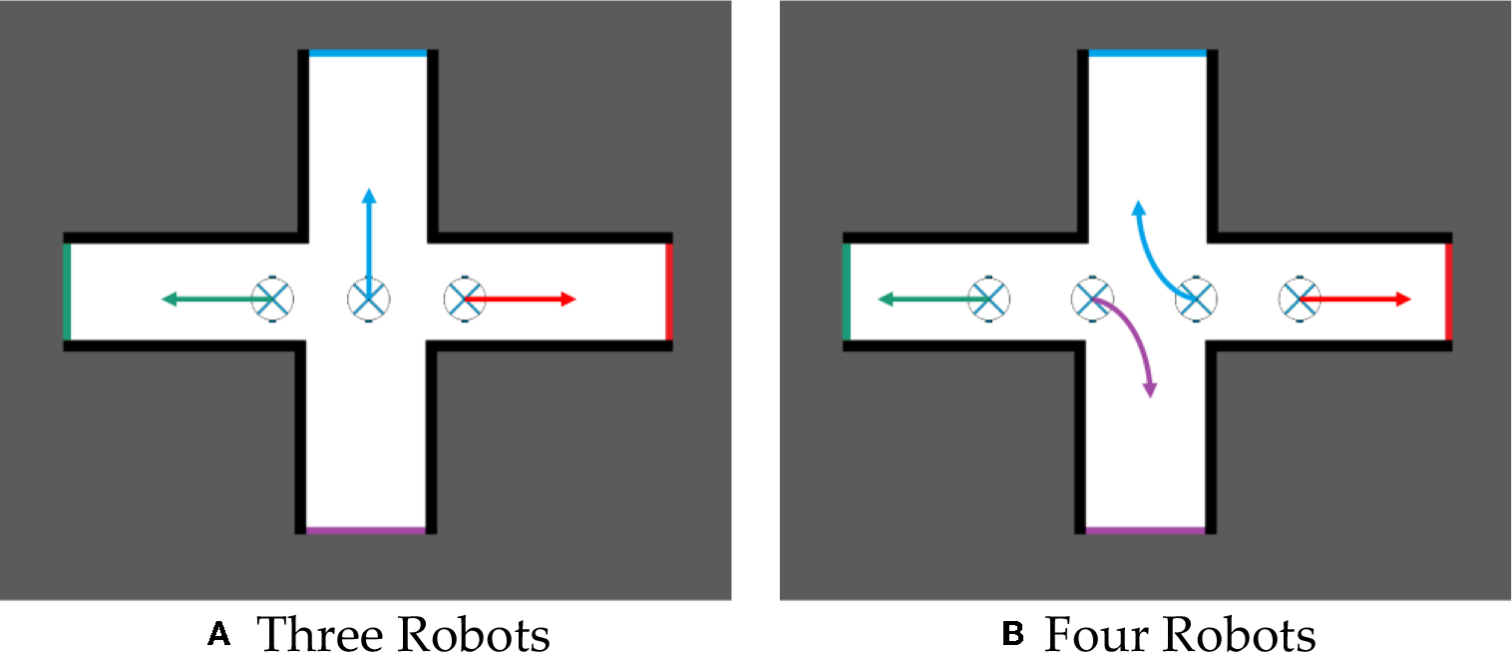
Multi-robot task allocation. **(A)** Three robots. **(B)** Four robots.

The Hungarian algorithm is used to allocate frontiers to robots, which can maximize the efficiency of multi-robot cooperative autonomous exploration. For example, in Figure 4, three or four robots build a map while exploring an unknown environment together. If they reach a crossroads, the target points assigned to each robot will make them tend to explore the environment along different corridors.

In real-life situations, multi-robot systems often operate in complex and hostile environments that can lead to robot failure or breakdown. In order to ensure the continued operation of the multi-robot system, it is necessary to propose a self-healing cooperative autonomous exploration method that can detect and recover from robot failures. This method can significantly improve the robustness and maintenance efficiency of the system.

**Figure 5:**
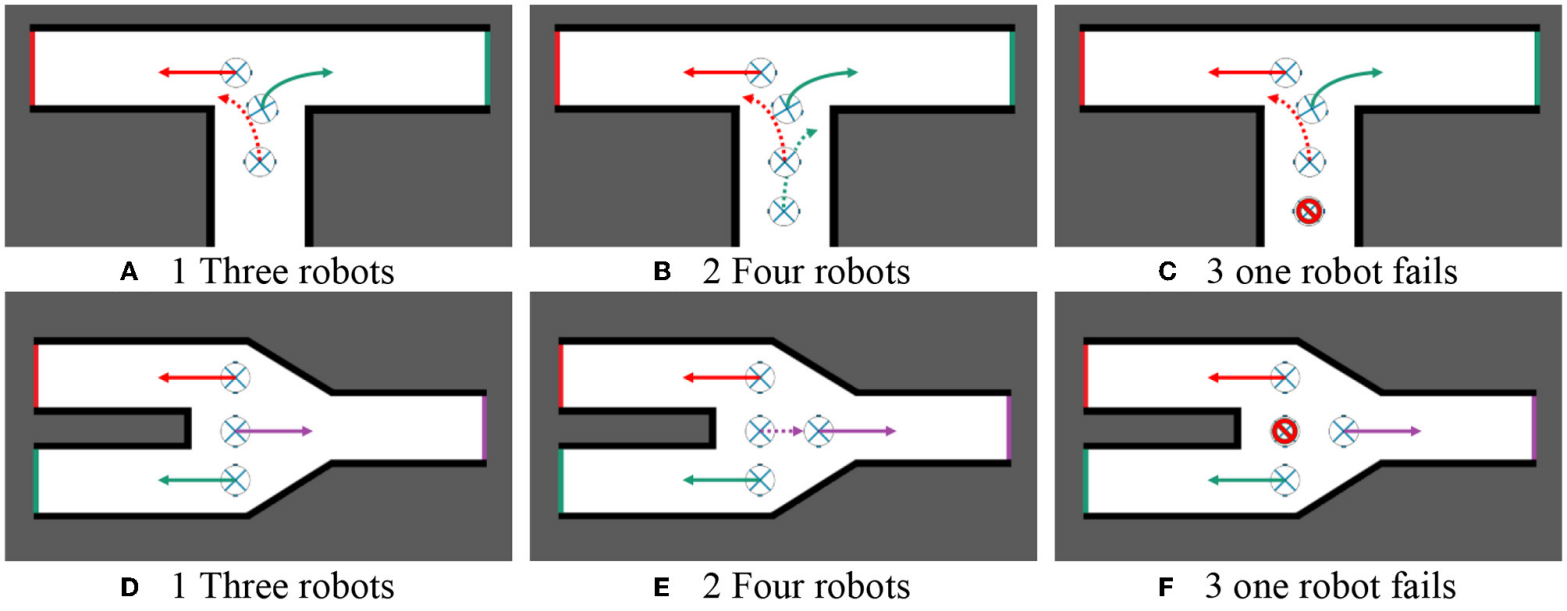
Self-healing cooperative autonomous exploration. **(A)** Three robots. **(B)** Four robots. **(C)** One robot fails. **(D)** Three robots. **(E)** Four robots. **(F)** One robot fails.

The main idea is to check whether robots are working properly before assigning tasks to robots. If the robot does not work, it will no longer assign tasks to the robot, but continue to assign tasks to other working robots and carry out path planning. Figure 5 shows two hypothetical scenarios. When four robots cooperate to explore the environment, one robot fails to work due to some condition, but the self-healing cooperative autonomous exploration method can still effectively assign tasks for robots in case of robot failure.

### 3.3. Geometric optimization

After obtaining the assigned goals of robots, it is necessary to use the path planning algorithm to plan the path of each robot to its goal. Up to now, many scholars have proposed a variety of efficient path-planning algorithms, such as Dijstra (Liu et al., 2021), A^*^ (Hart et al., 1968), RRT (LaValle and Kuffner, 1999), and so on. This paper makes some optimization on the path, which can shorten the path and make the path smoother to a certain extent. Taking the optimization of Informed RRT^*^ (Karaman and Frazzoli, 2011; Gammell et al., 2014) as an example, the path optimization algorithm proposed in this paper can be applied to all the above algorithms. Algorithm 3 presents a geometric optimization algorithm. The algorithm that follows the pseudo-code shown in Algorithm 3 is then explained, the Input is an initial solution path $P^{I}=\left\{p_{1},p_{2},\cdots \;,p_{n}\right\}$. The algorithm describes two cases. In the first case (i is odd), the method takes the route *P* from *p*_*start*_ to *p*_*goal*_. In the other case (i is even), the waypoints of *P* are arranged in the reverse direction from *p*_*goal*_ to *p*_*start*_. Apart from that, the points *P*_*pre*_ and plast record, respectively, the last collision-free connection point and the present point that has to be investigated. Next, it is determined if there are no collisions along the direct path between *P*_*pre*_ and *p*_*j*_. As there is no conflict on the direct line from *P*_*pre*_ to *p*_*j*_, *P*_*cur*_ is updated if flag is true. If flag is false, the line connection from *P*_*pre*_ to *P*_*cur*_ is collision-free. As a result, we acknowledge *P*_*cur*_ as part of the solution path and begin looking for a collision-free link from *P*_*cur*_ again. The algorithm is shown in Table 3.

**Table 3:** Geometric optimization.

**Algorithm 3: Geometric optimization**
Input: Initial Path $P^{I}=\left\{p_{1},p_{2},\cdots \;,p_{n}\right\}$
Output: Final Path *P*^*F*^
1: for *i* = 1 ⋯ 2^*^M do :
2: Initialize: *P*^*F*^ = [];*P*_*pre*_ = *p*_1_; *P*_*cur*_ = *p*_1_
3: for *j =* 2⋯|*P*^*I*^| do :
4: if *L*(*P*_*pre*_, *p*_*j*_)∩ℂ_*obs*_ = ∅ do :
5: *p*_*cur*_ = *p*_*k*_
6: else
7: *j = j–*1
8: *p*_*pre*_ = *p*_*cur*_
9: *P*^*F*^*.push*(*p*_*cur*_)
10: if *p*_*cur*_ = *p*_|*P*|_ do :
11: end for
12: ∀*k = 1*⋯|*P*|:*p*_*i*_ = *p*_|*P*|+1 − *k*_
13: *P*^*I*^ = *P*^*F*^
14: end for
15: return *P*^*F*^

## 4. Simulation and experiments

### 4.1. Simulation on path planning

**Figure 6:**
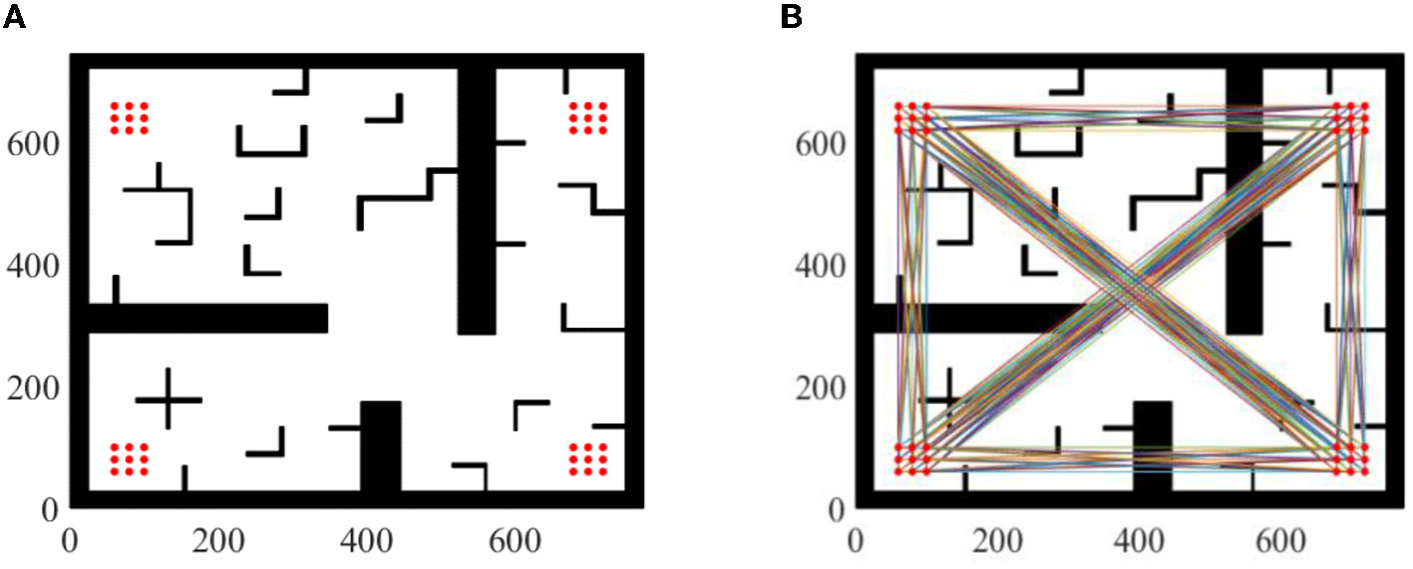
Experiment setup. **(A)** 36 sampling points. **(B)** 576 possible scenarios.

To avoid differences in results due to random sampling, it is necessary to analyze the results of multiple paths. The importance of analyzing the results of multiple paths can be demonstrated through synthetic experiments, as shown in Figure 6. In this experimental environment, 36(4^*^9) sampling points were obtained by sampling the four corners of the environment. Each sampling point in the four corners was combined with each sampling point in the other corners as the starting and ending point of path planning. However, points in the same corner were not combined with each other, resulting in 576($C_{4}^{2*}$9^*^9) possible scenarios.

**Figure 7:**
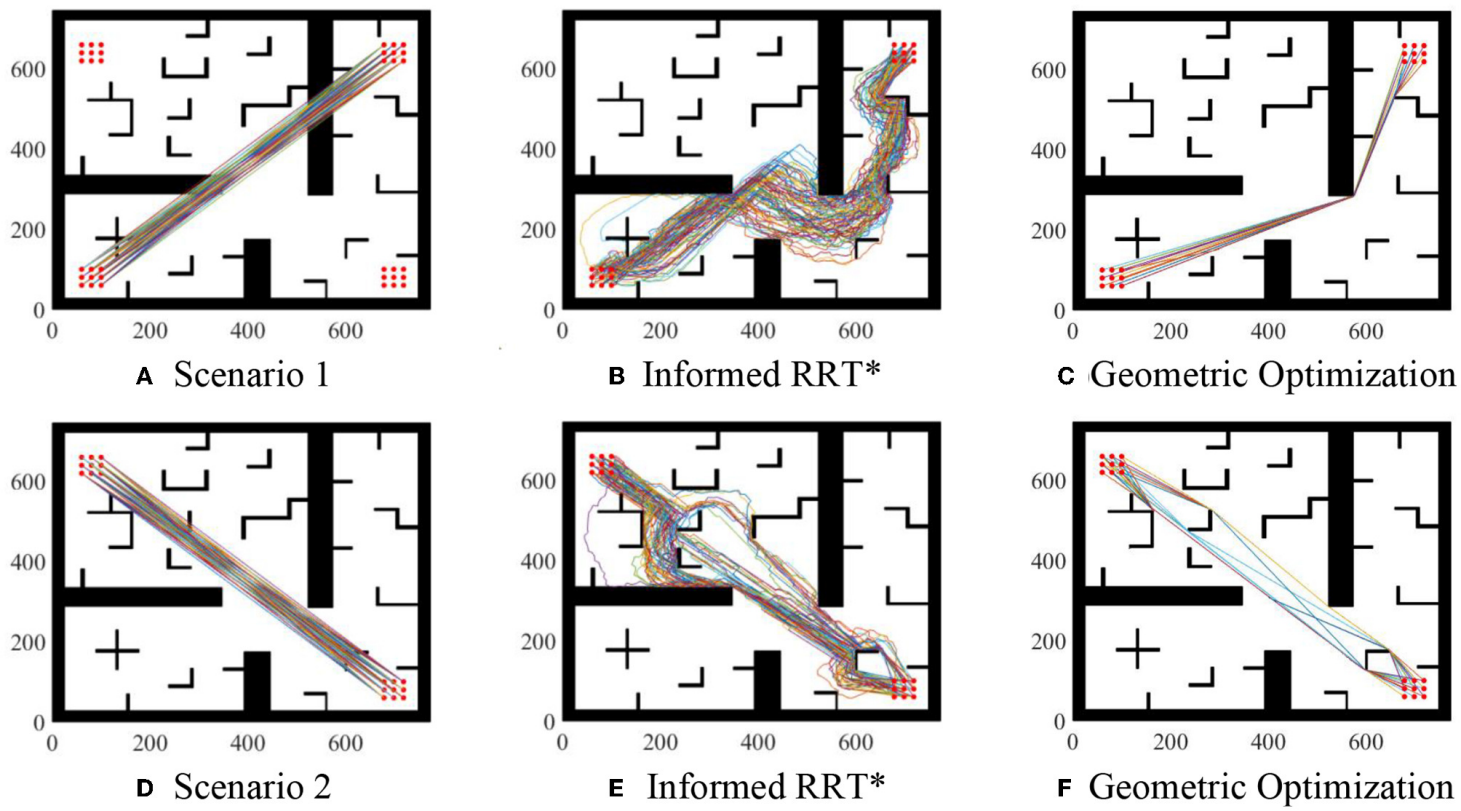
Geometric optimization. **(A)** Scenario 1. **(B)** Informed RRT*. **(C)** Geometric optimization. **(D)** Scenario 2. **(E)** Informed RRT*. **(F)** Geometric optimization.

**Figure 8:**
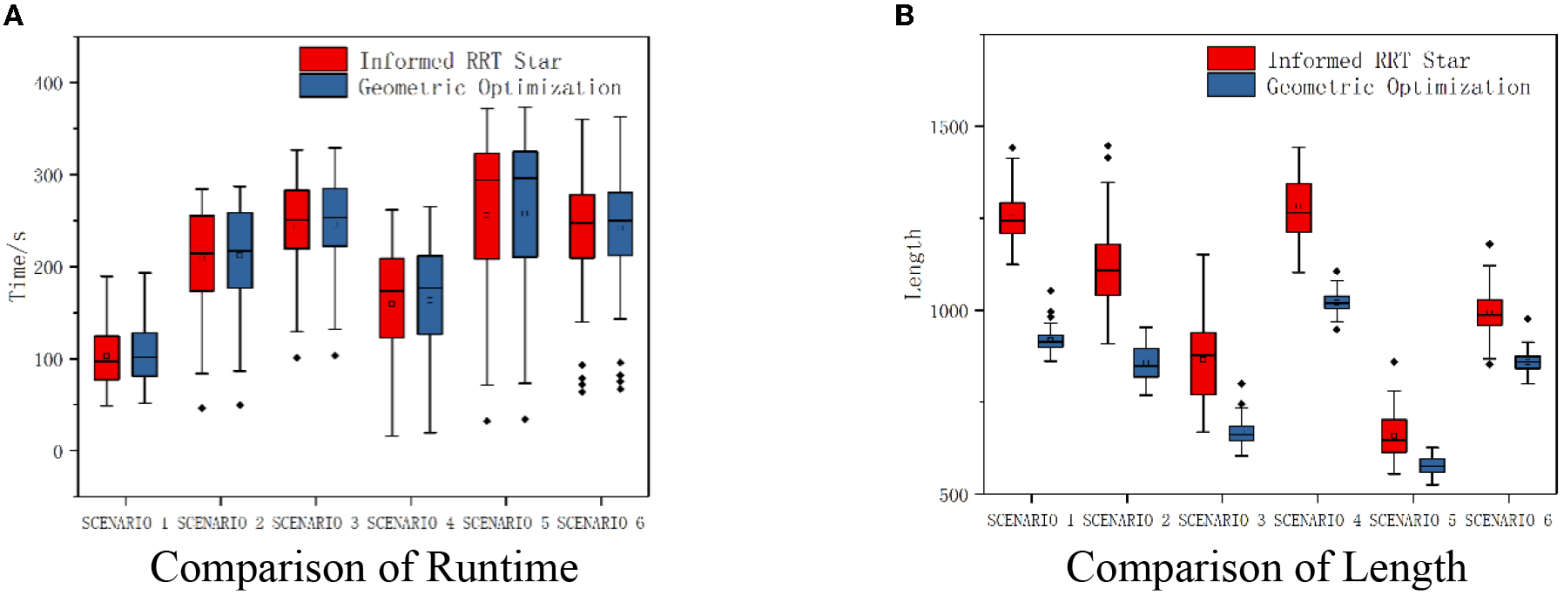
Experiment comparison. **(A)** Comparison of runtime. **(B)** Comparison of length.

Parameter setting: maximum sampling number is 8,000 and sampling step length is 10. Take two of these scenarios as examples, as shown in Figure 7. Compared with Informed RRT^*^ and geometric optimization, it is shown in the experiments that our method is more effective. The path optimization algorithm proposed in this paper linearizes the path repeatedly, which greatly shortens path length compared with initial paths. The running time of the two algorithms is almost the same. The average running time of algorithm 2 will be about 2% more than that of algorithm 1, but the average length of algorithm 2 will be about 30% shorter than that of algorithm 1, as shown in Figure 8.

### 4.2. Simulation on cooperative exploration

**Figure 9:**
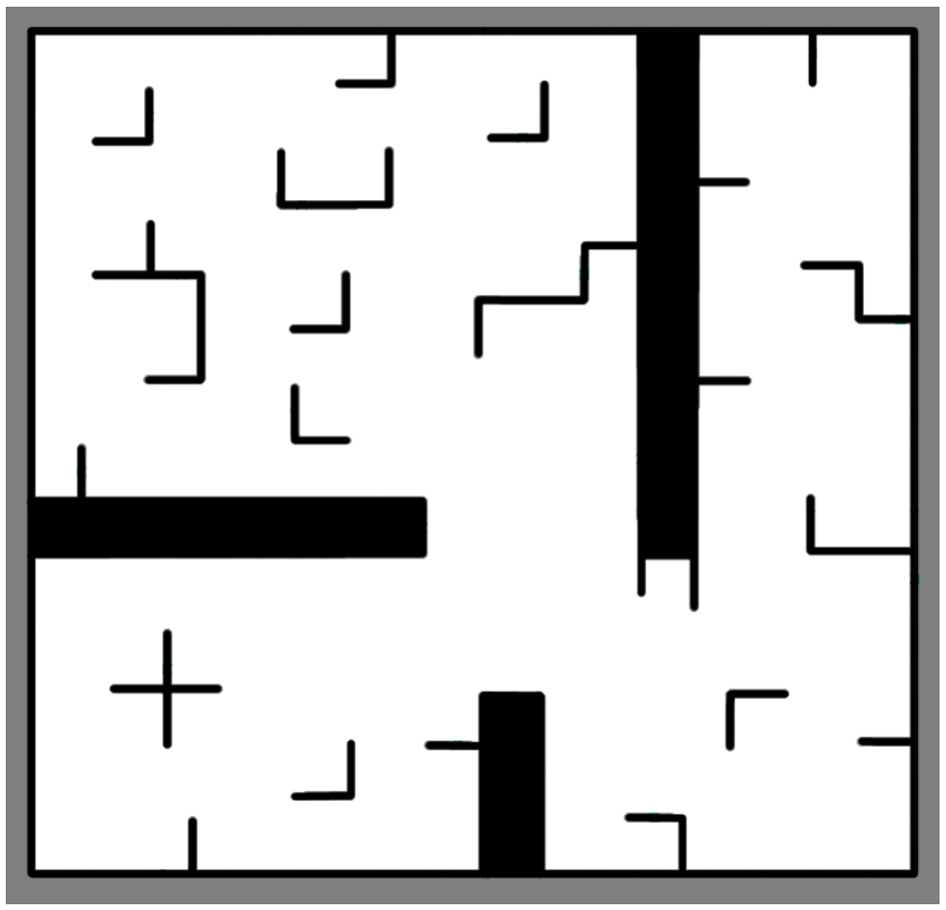
Environments using for simulation on cooperative autonomous exploration.

Our method was evaluated in a two-dimensional simulation environment. Figure 9 shows the test simulation environments used for experiments. The cooperative autonomous exploration method for multi-robot can complete the task of cooperative autonomous exploration, even some robots fail to work suddenly.

The environment has 186^*^193 grids (37.2 *m* ^*^ 38.6 *m*), including various obstacles, with various typical characteristics and strong representativeness. In the above environment, black indicates obstacles and white indicates that the area is a passable area. The radar detection range is 8 *m*, and the radar noise is Gaussian noise. Cooperative autonomous exploration will stop when more than 98% of environments are observed or frontiers do not exist.

To prove that the cooperative autonomous exploration method for multi-robot is feasible and reliable, compare our method with Yamauchi's method where robots navigate to their nearest frontiers separately (Algorithm 3 was adopted to plan path).

**Figure 10:**
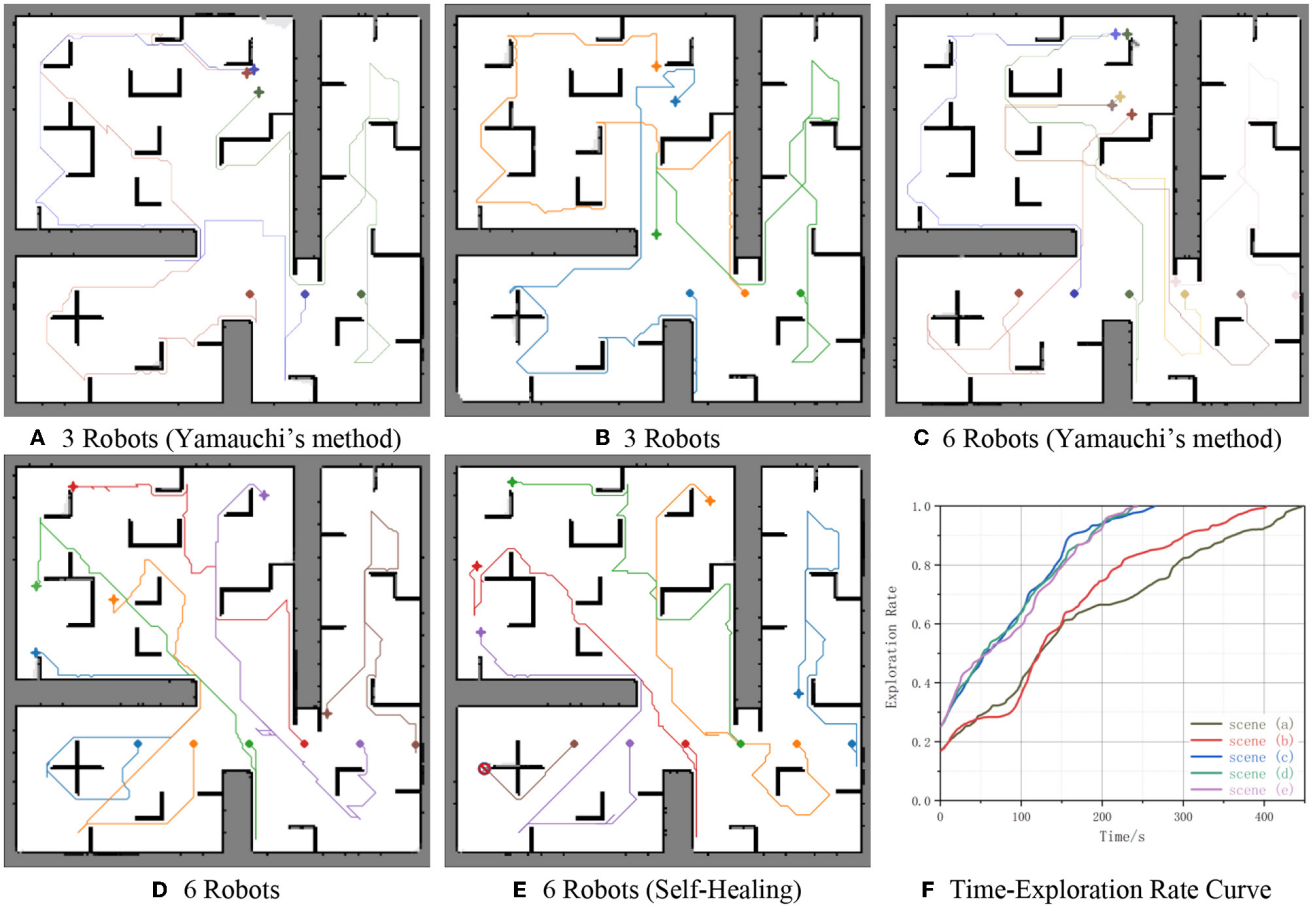
Simulation results. **(A)** 3 Robots (Yamauchi's method). **(B)** 3 Robots. **(C)** 6 Robots (Yamauchi's method). **(D)** 6 Robots. **(E)** 6 Robots (self-healing). **(F)** Time-exploration rate curve.

Figure 10 shows the occupancy grid maps obtained by multi-robot cooperative autonomous exploration, along with the robot moving paths and the results of five experiments. In the subgraph Figures 10A–E: Diamonds indicate initial positions of robots; Stars indicate Final positions of robots; Colorful lines indicate paths of robots from initial positions to final positions; The red slash icon indicates that robots do not work for some reason and cannot move when reaching the position; Black grids indicate obstacles and robots cannot pass; White grids indicate that the area is passable; Gray grids indicate that the area is unknown. In the subgraph Figure 10F: The horizontal axis shows time in seconds; The vertical axis shows exploration rate; Curve (*x*) indicate curve of exploration rate over time in the subgraph(*x*).

The experiments involved three or six robots exploring the environment together. The results demonstrate the effectiveness and efficiency of the multi-robot cooperative autonomous exploration method in generating accurate and comprehensive maps of the environment. Figures 10A, B shows three robots exploring the environment together, initial coordinates of robots from left to right are (22 *m*, 11 *m*), (27 *m*, 11 *m*), and (32 *m*, 11 *m*), respectively. Figures 10C–E shows six robots exploring the environment together, initial coordinates of robots from left to right are (12 *m*, 11 *m*), (17 *m*, 11 *m*), (22 *m*, 11 *m*), (27 *m*, 11 *m*), (32 *m*, 11 *m*), (37 *m*, 11 *m*), respectively. However, the robot whose initial position is (12 *m*, 11 *m*) in Figure 10E does not work for some reason suddenly when time = 50s. This highlights the importance of self-healing strategies for multi-robot systems, which can recover from robot failures to ensure the continued operation of the system.

It can be seen from Figure 10, the environment is fully explored. And our method explores more than 98% of the unknown environment, as confirmed by the Time-Exploration Rate Curve in Figure 10. Moreover, the comparison of the two groups of curves Figures 10A–D shows that the proposed method is more efficient than Yamauchi's method, with a reduction in time of 27 s (11.34%) and 43 s (10.67%), respectively. This demonstrates the effectiveness of the proposed method in optimizing the exploration path.

In addition, the results also demonstrate the reliability of the proposed method. Even if one of the robots fails to work, the cooperative autonomous exploration method is still able to explore more than 98% of the unknown environment. Comparing two curves Figures 10D, E, the exploration rate has changed little when only one of the six robots fails to work. The change range of the exploration rate is <5% at the same time, and the completion time difference is no more than 5 s (2.10%), this shows our method is reliable. This indicates that the proposed method is reliable and able to adapt to unexpected situations, such as robot failures.

### 4.3. Experiment

To further validate the effectiveness of the proposed algorithm for cooperative autonomous exploration, the algorithm was tested in a more realistic simulation environment. ROS is a systematic framework for robot development and provides a modular and flexible architecture for building robot applications. Gazebo is an open-source 3D robot simulation software that allows for realistic simulation of robot behavior and interaction with the environment. Together, ROS and Gazebo provide a powerful toolset for developing and testing robotic systems, and this combination enables developers to rapidly prototype and test their robot designs in a virtual environment before deploying them in the real world. The robot we used was a standard robot development platform: Scout-Mini, which can be equipped with laser radar and directly controlled via the speed topic. Laser radar can detect objects as close as 0.3 *m* and as far as 6 *m* away, and Gaussian noise is added to the sensor data. The raw data from the laser radar is sampled at a range of 360, which means that it can capture a complete view of the surrounding environment.

The experiment involved two robots working together to perform cooperative autonomous exploration tasks. Use Cartgrapher algorithm to build the map, and update the merged map through the updating formula of an occupancy grid map by multiple robots (Hess et al., 2016). Use DWA (Dynamic Window Approach) algorithm to control the robots, DWA algorithm is a local obstacle avoidance algorithm and converts the position control into the speed control (Seder and Petrović, 2007).

**Figure 11:**
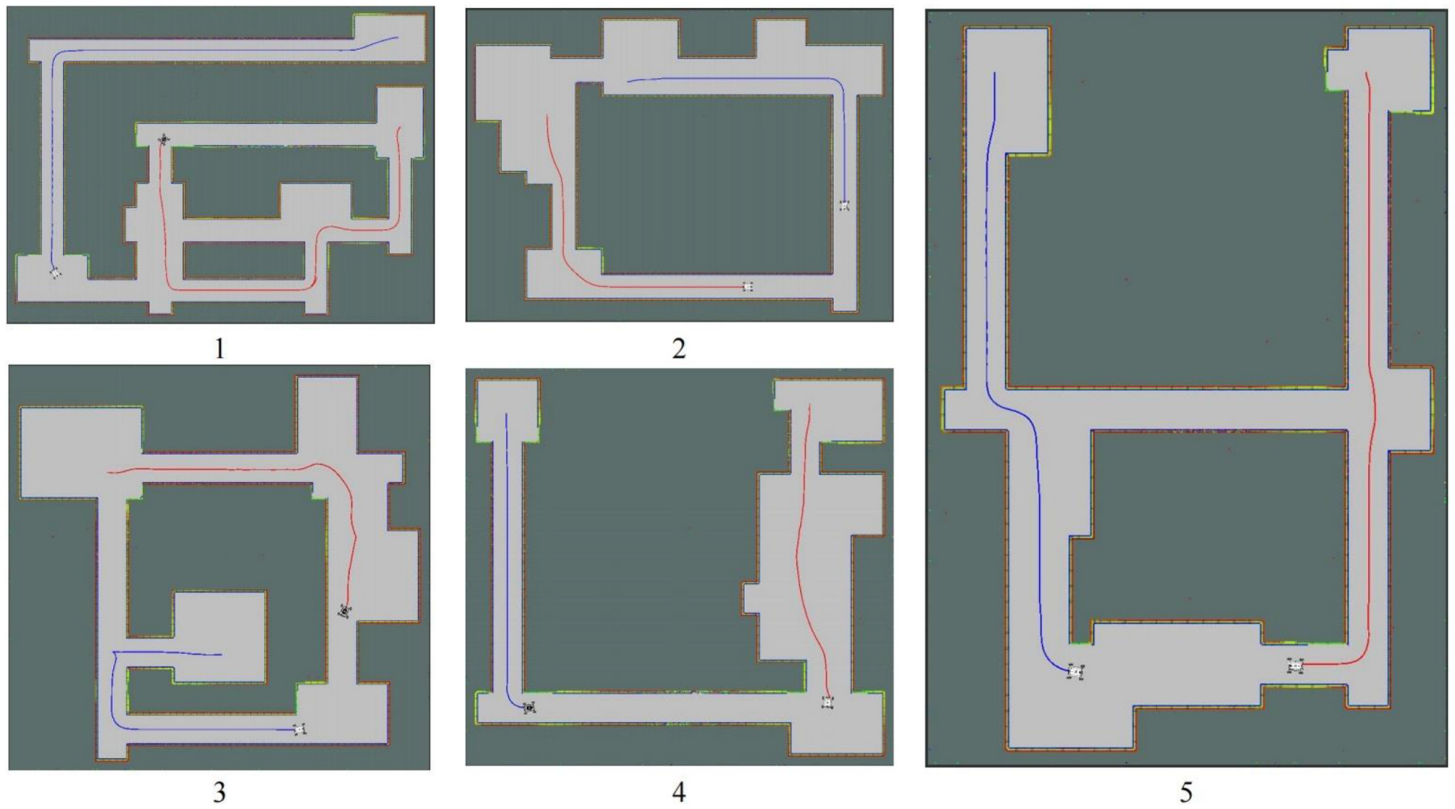
Environments using for experiments. The blue line represents the first robot's pathway; The red line represents the second robot's pathway.

Figure 11 shows the results of cooperative exploration in five different scenarios, and cooperative autonomous exploration experimental data is shown in Table 4. Despite the noise of the laser radar, the maps obtained still accurately represented the basic characteristics of the environment. The results demonstrate that the task of collaborative exploration can be completed efficiently, using the proposed method. Additionally, from the exploration routes of the two robots in each scenario in Figure 11, it can be observed that the two robots are assigned to different areas to explore, which helps to improve the exploration efficiency. By dividing the area into different groups and assigning each robot to explore a specific area, the exploration task can be completed more efficiently and in less time.

**Table 4:** Cooperative autonomous exploration experimental data.

**Scenario**	**Robot 1 (unit :** ***m*****)**			**Robot 2 (unit :** ***m*****)**		
	**Start (X, Y)**	**End (X, Y)**	**Length (** ***l*** **)**	**Start (X, Y)**	**End (X, Y)**	**Length (** ***l*** **)**
1	(26, 19)	(2.8, 4.4)	36.4	(26, 13)	(10, 11.6)	35.9
2	(10, 15)	(23.6, 7.1)	21.3	(5, 13)	(17.7, 2.1)	21.5
3	(11, 6)	(15.5, 2.1)	19.3	(5, 15.5)	(17.4, 8.3)	19.7
4	(2, 18)	(2.9, 3)	15.5	(17.5, 18.5)	(18.3, 3.1)	15.6
5	(26, 17)	(3.4, 14)	24.4	(26,3)	(3.6, 5.7)	24.9

Moreover, by comparing the length of the two robots' routes in Table 4, it can be seen that the length of the routes is basically the same. This indicates that the distribution of tasks is balanced in the exploration process. By balancing the tasks assigned to each robot, the overall exploration efficiency can be improved.

Additional single-robot autonomous exploration experiments were conducted to highlight the efficiency of multi-robot cooperative autonomous exploration. Experiments were conducted in the same five scenarios mentioned above, but only using the first robot to explore unknown environments. Single-robot autonomous exploration experimental data is shown in Table 5, the efficiency in the table refers to the path length of single-robot autonomous exploration divided by the path length of the first robot in multi-robot cooperative autonomous exploration. According to the data in Table 5, it can be concluded that collaborative autonomous exploration is more efficient than single-robot autonomous exploration. The efficiency in the above five scenarios can reach a minimum of 182.4% and a maximum of 449.0%, with an average efficiency of 296.1%.

**Table 5:** Single-robot autonomous exploration experimental data.

**Scenario**	**Robot (unit:** ***m*****)**			
	**Start (X,Y)**	**End (X,Y)**	**Length (** ***l*** **)**	**Efficiency**
1	(26, 19)	(25.3, 11.4)	106.5	292.6%
2	(10, 15)	(5.6, 13.1)	66.1	310.3%
3	(11, 6)	(5.5, 15.1)	47.5	246.1%
4	(2, 18)	(17.9, 18.3)	69.6	449.0%
5	(26, 17)	(26.4, 3.7)	44.5	182.4%

To further validate the effectiveness of the proposed method for cooperative autonomous exploration in a real-world scenario, the algorithm was tested using two real robots with communication supported through a router.

**Figure 12:**
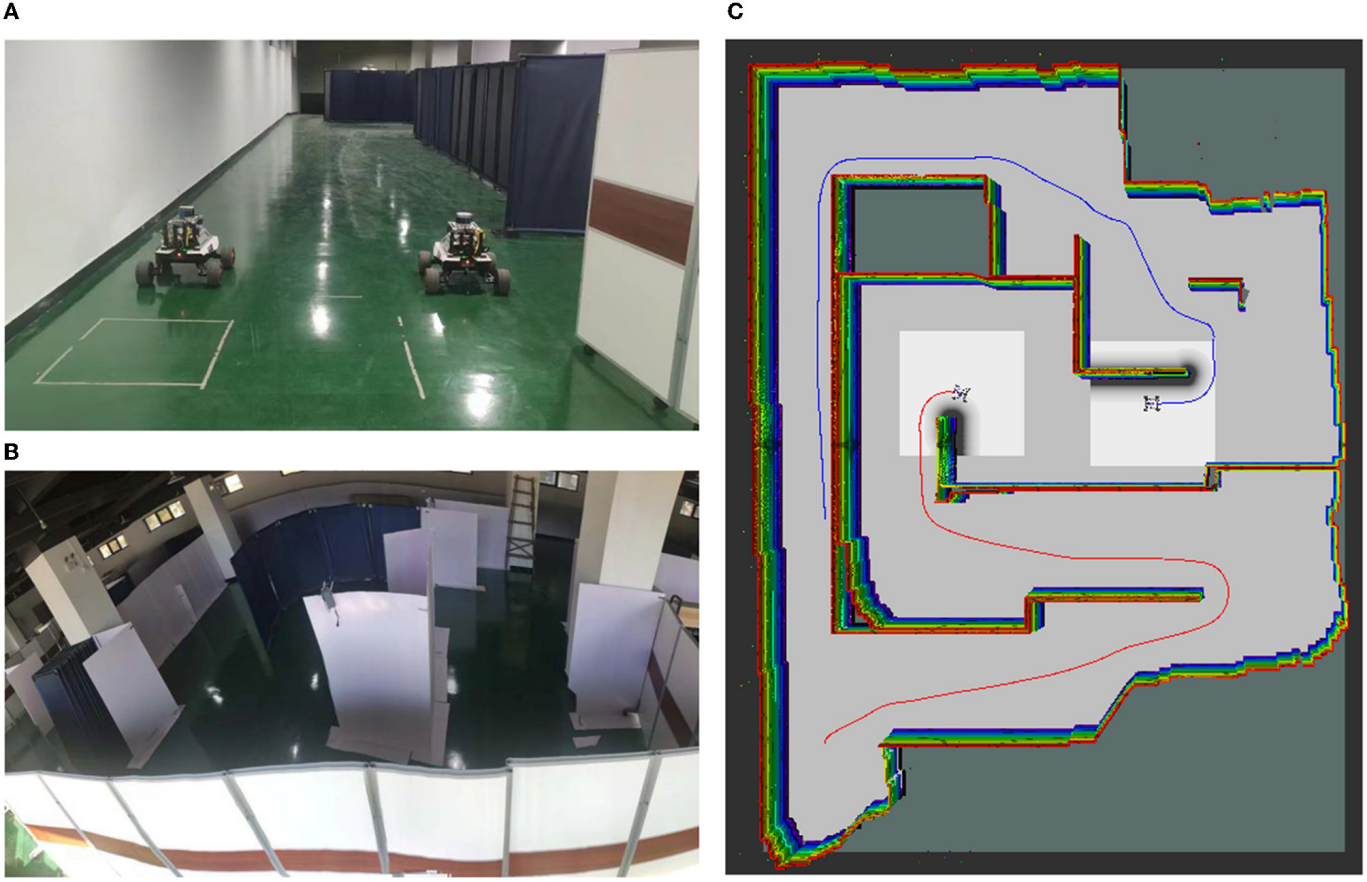
Real robot: **(A)** Two robots: Scout-MINI; **(B)** The real Scenario 1 used in the experiment (15 *m* * 20 *m* in dimensions); **(C)** The green line represents the first robot's pathway; The red line represents the second robot's pathway.

The mobile robot platform used in the experiment is All Terrain UGV: Scout Mini, as shown in Figure 12A, with a robot size of 126 mm^*^580 mm^*^245 *mm*, a maximum movement speed of 3 *m*/*s*, and an overall weight of 23 *Kg*. The mobile robot is equipped with Velodyne LiDAR: VLP-16, and the VLP-16 has an effective ranging range of 6 *m*, with a 360 horizontal field of view angle and an adjustable range of up and down 15. The upper computer is the XAVIER edge computing development system (XAVIER GE Kit). The odometer and IMU (Inertial Measuring Unit) provide locating information. The communication between equipment is supported through the router, and the Jetson Nano is used as the master control device to process data and run our algorithm.

The experiment was conducted in a warehouse environment, as shown in Figure 12B. The presence of baffle walls in the environment greatly increased the complexity of the area, making cooperative autonomous exploration more difficult. However, the proposed method was able to effectively explore the environment. Figure 12C shows the occupancy grid obtained during completing the autonomous exploration task on the real robot. By comparing our experimental environment with the final results obtained through cooperative autonomous exploration, it can be found that the environmental features are completely established. Our method explores all areas in unknown environments and realizes the autonomous exploration task of an unknown real environment without any prior knowledge.

The experimental results demonstrate that the proposed cooperative autonomous exploration method is able to effectively and efficiently explore unknown environments in real-world scenarios. By using multiple robots working together and communicating with each other, the method is able to balance the distribution of tasks, and optimize the exploration path, resulting in high efficiency and accuracy in cooperative autonomous exploration.

## 5. Conclusion and discussion

A cooperative autonomous exploration method for multi-robot is proposed in this paper. The method involves three main components: frontiers grouping and clustering, task allocation and path planning. Frontiers grouping and clustering enables the robot to efficiently explore the environment by grouping similar frontiers and avoiding redundant exploration. Task allocation involves assigning tasks to the robot based on the requirements of the exploration mission. The tasks are assigned based on a centralized control system, which ensures that the robot's actions are coordinated and optimized for efficiency and effectiveness. Another important aspect of multi-robot cooperative autonomous exploration is the ability to handle robot failure. This can be achieved through the use of proposed self-healing algorithms in this paper, where the remaining robots re-allocate the failed robot's tasks and continue the exploration task. Path planning is the third component of the method. It involves generating optimal paths for the robot to follow in order to complete its assigned tasks. The paths are designed to minimize the distance traveled by the robot and ensure that it reaches its targets efficiently. The experimental results show that the proposed method can achieve exploration of the environment while minimizing the exploration time and route length. Overall, the experimental results support the conclusion that the cooperative autonomous exploration method is effective and efficient in completing the task of exploring unknown environments.

The work in this paper is based on frontiers, adopts the method of information theory, designs the corresponding effect function to evaluate target points, and selects the next best target point. Traditional methods have achieved some results, but need to detect frontiers on the global map, detection efficiency is closely related to complexity of the scenario and have significantly positive correlation differences in decision-making efficiency with different complexity. Autonomous exploration method based on reinforcement learning can shorten the computation time of the decision-making process and improve the efficiency of decision-making, but the robot may cannot find an effective action. In the future, we will adopt a hybrid method to improve the autonomy and intelligence of robots, which combines learn-based methods with traditional methods.

## Data availability statement

The original contributions presented in the study are included in the article/supplementary material, further inquiries can be directed to the corresponding author.

## Author contributions

All authors listed have made a substantial, direct, and intellectual contribution to the work and approved it for publication.
